# Transosseous Suture Fixation of True 4-part Valgus Impacted Fractures of the Proximal Humerus: Clinical and Radiological Outcome in 49 Patients

**DOI:** 10.2174/1874325001812010041

**Published:** 2018-02-08

**Authors:** Andreas Panagopoulos, Irini Tatani, Seferlis Yannis, Bavelou Aikaterini, Antonis Kouzelis, Minos Tyllianakis, Panayotis Dimakopoulos

**Affiliations:** Shoulder & Elbow Department – University Hospital of Patras, Patras, Greece

**Keywords:** Humeral head, Transosseous suturing, ORIF, 4-part fracture, Valgus impaction, Constant score

## Abstract

**Background:**

The valgus-impacted (VI) 4-part fractures are a subset of fractures of the proximal humerus with a unique anatomic configuration characterized by a relatively lower incidence of avascular necrosis after operative intervention.

**Objectives:**

The present study reports the midterm clinical and radiological results of a large series of consecutive patients with 4-part VI fractures treated with a minimal invasive technique of internal fixation.

**Methods:**

Over a ten-year period (2004-2014), we treated 56 patients with a true 4-part valgus impacted fracture of the proximal part of the humerus. Four patients were lost to follow-up and three died, leaving 49 patients (33 female, 16 males, average age 60,1 years) available for the study. Fracture fixation was achieved through the lateral transdeltoid approach with transosseous suturing of the tuberosities to each other, to the metaphysis and to the articular part of the humeral head avoiding gross disimpaction of the humeral head from the valgus position. Functional outcome assessment was performed using the parameters of the Constant-Murley score within a mean follow up period of 43,8 months (range, 24 to 115 months).

**Results:**

All fractures were united within the first 3 months except one that showed late displacement and finally nonunion. The median Constant score was 81,7 points and the functional score in comparison with the unaffected shoulder was 86.2%. There were three patients with total Avascular Necrosis (AVN) of the head revised to hemiarthroplasty. The nonunion case was revised to reverse shoulder arthroplasty 12 months after surgery. In five cases, absorption of the greater tuberosity was noted in the last radiographic control without any serious consequences to the shoulder function.

**Conclusion:**

Advantages of this minimally invasive technique can be summarized as shorter operative time, no use of hardware, minimal soft tissue damage, low incidence of avascular necrosis, stable osteosynthesis with “tension band effect” and adequate rotator cuff repair allowing for early joint motion.

## 1 INTRODUCTION

Accounting for approximately 14% of all humeral head fractures, the 4-part valgus-impacted fracture was first featured by Jacob *et al*. [1] and was considered as a subtype of proximal humeral fractures, in which the articular segment is impacted into the metaphysis, causing spread of the greater and lesser tuberosities thus creating a fracture line through the anatomical neck, with minimal or zero disruption of the posteromedial hinge. In 2002, Neer CS [2] updated his 4-segment classification of proximal humeral fractures and included the 4-part valgus impacted fracture, as a borderline lesion (type A) in the continuum of the lateral displacement of the head that progresses from those with minimal displacement to the valgus impacted type and then on to the true 4-part fracture (lateral fracture-dislocation,

type B). The AO/OTA classification in contrast contains the 4-part VI fracture in the subgroup 11-C1.1 (slight displacement) and 11-C2.1 (marked displacement) [3]. Robinson *et al* [4] described the injury in terms of anatomical features and suggested a grading system from stage 1 (undisplaced humeral head) to stage 2 (valgus impaction and lateral head translation) to the more severe stage 3 (denuded humeral head with high risk of osteonecrosis).

Treatment options for this unique fracture range from non-operative care to internal fixation and shoulder arthroplasty. Court Brown *et al*. [5] reported 80% good or excellent results of conservative treatment in a large population of 125 patients with minimal displaced fractures; however, functional outcome was less predictable in the more severe types. For younger patients operative treatment is highly recommended, because the deforming forces of rotator cuff (RC) tendons can produce secondary tuberosity displacement leading to subacromial impingement, blocking of flexion and external rotation and early RC arthropathy and osteoarthritis [4, 5].

Locking plates represent a significant advancement in the treatment of these injuries as they can provide more secure fixation in osteoporotic fractures, but recent systematic reviews have shown very high rate of complications (varus malunion, screw perforation, subacromial impingement and AVN) [6, 7]. Brorson *et al*. [8] recommended in their systematic review (2012) to avoid the routinely use of locking plates in AO/OTA Type C fractures (including VI types) as they found an overall reoperation rate ranged from 6 to 44%. Even with the use of minimal invasive percutaneous plating techniques (MIPO) the complication rate in 4-part fractures can be as high as 19% [9].

Several other authors [10-17] have proposed closed or open reduction and fixation of the 4-part VI fracture using less invasive fixation techniques such as isolated sutures, intramedullary pins, tension band wiring, screws, Kirschner wires or combination of these. Panagopoulos *et al* [18] reported recently (2016) a systematic review of least possible fixation techniques (LPFT) in 4-part valgus impacted fractures and found 12 eligible studies included 190 VI fractures in 188 patients. The overall AVN rate was 11%, the re-operation rate 3.7% and a good functional outcome (Constant Score >80) was reported in 9/12 studies.

The present study reports the midterm clinical and radiological results of a large series of consecutive patients with 4-part VI fractures treated with a minimal invasive technique of internal fixation.

## 2 MATERIALS AND METHODS

During a ten-year period (2004-2014), a consecutive series of 427 patients underwent surgical treatment for displaced fractures of the proximal humerus in our Department. Internal fixation with transosseous sutures was applied in 138 patients; 56 of them had sustained a true 4-part valgus-impacted fracture. Eligible for inclusion into the study were considered those patients who were medically fit to receive general anesthesia and mentally alert to cooperate with the prolonged rehabilitation protocol. Written informed consent was obtained by all participants for prospective data collection, follow up exams and publication of the clinical and radiological results in international journals. Ethical approval was not necessary, according to our committee, for this type of follow up study as the same established and already published surgical treatment was applied to all patients.

**Fig. (1):**
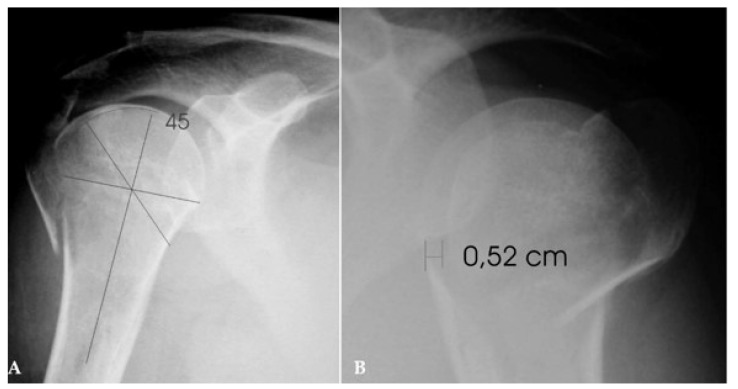
Estimation of the valgus impaction angle (**A**) and the medial or lateral translation of the head in respect to the diaphysis (**B**) using the measuring tools of the PACS system.

All patients had sustained a fresh or not older than one month, fracture of the proximal humerus with true valgus impaction: the later was defined as the angle between the fracture plane of the articular segment and the axis of the humeral shaft and was accurately measured together with the length of posteromedial hinge, in the initial anteroposterior radiograph in zero rotation; additional CT-scan images were obtained when it was necessary (Fig. **1**). The radiological criteria for considering a fracture as amenable to osteosuture fixation were: a) loss of posteromedial hinge integrity, either medially or laterally, no greater than 10 mm, b) valgus impaction angle up to 45 degrees, c) displacement or angulation of the greater tuberosity by > 5 mm or 45 degrees in respect and finally d) presence of adequate metaphyseal head extension by minimum 10 mm.

Four patients were lost to follow-up and three died from causes unrelated to the fracture, leaving 49 patients for follow up evaluation. There were 33 women and 16 men with an average age of 60,1 years (range, 21-82 years) at the time of injury (Table **1**). Thirty-six patients sustained a fracture upon a standing or lower height fall, 2 upon falling from heights > 2m and 11 after involving in a motor vehicle accident. Associated injuries were present in seven patients (14.2%) while four patients had sustained old injuries (range, 16 to 30 days) and were referred to our Department by other hospitals. The right shoulder was affected in 32 cases and the left in 17. There were no clinically detectable neurovascular deficits preoperatively. Thirty-six patients had been regularly employed prior to the injury: twenty-two had a sedentary job, and fourteen performed manual work.

### 2.1 Surgical Technique

**Fig. (2):**
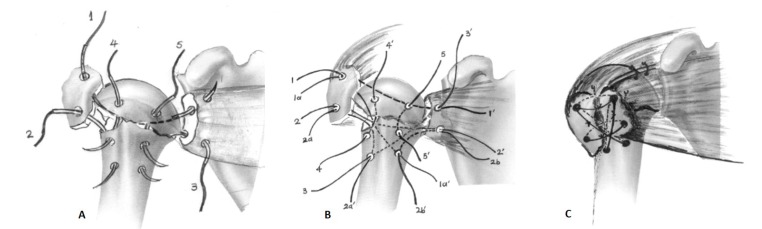
Schematic representation of our surgical technique: **A**. initial passage of heavy transosseous sutures to both tuberosities, to the articular segment and at both sides of the diaphysis, **B**. Additional sutures are passing after provisional reduction in a special numerical order to secure the tuberosities not only to each other but both to the articular part of the head in a cruciate manner, **C**. the final knotting results to a stable fixation with “tension band effect”.

Under general anesthesia the patient was placed in the “beach-chair” position with at least 70^o^ of flexion at the waist and two folded sheets behind the scapula to bring the shoulder girdle forward in order to facilitate access to glenohumeral joint. The entire upper extremity was prepared and draped in a manner to allow full and unrestricted arm positioning during the procedure. The anterolateral trans-deltoid approach was employed to all patients as it provides adequate access to the humeral head. The fracture pattern constantly consistent of a superiorly facing humeral head and both the tuberosities splayed on either side of it, having more than 5 mm of displacement in all cases. A subsequent tear in the rotator cuff, which was propagated through the rotator interval, was confirmed in 27/44 cases, requiring repair with non-absorbable sutures. After gently separation of the fracture lines with a periosteal elevator, two or three pairs of number-5 Ethibond sutures were inserted in each tuberosity, in the articular margin of the head fragment and in both sides of the diaphysis; in young patients, this was facilitated with a 2.7 mm drilling. The fracture was fixed thereafter by pulling down the tuberosities along to the diaphyseal axis, just below the top of the head part (which is slightly elevated) and tied up not only the tuberosities to each other but also to the articular fragment and to the medial and lateral side of the diaphysis, in a “tension band manner” (Fig. **2**). The surgical technique of fixation and the sequence of suture passing and knot tying have been previously published by the senior authors [19, 20]. Stability of the fixation was checked thereafter through the normal range of motion of the shoulder for any pathological movement between the shaft and the head. Suction drain placement was usually not necessary. The deltoid flaps and the subcutaneous tissue were re-approximated using absorbable sutures, and the skin intracutaneously. A Velpau dressing converted to a simple shoulder sling at the second postoperative day secured the arm to the chest wall.

### 2.2 Rehabilitation Protocol

A closely monitored 3-phase rehabilitation program was given to all patients initially consisted of pendulum exercises starting on the 2^nd^ postoperative day until the 2^th^-3^th^ postoperative week. The second phase included passive assisted exercises in the supine position as the patient was trying to reach the bed, supporting his injured shoulder by the healthy arm or special designed sticks. Until the 7^th^-8^th^ postoperative week, forward elevation and external rotation were performed in the supine position while internal rotation in the standing one with the aid of sticks. As the union progress was completed, active exercises using gradually increased weights (starting from 1 kilo) were administered until the 12^th^ postoperative week. If the patient was capable to forward elevate 2-3 kilos in the supine position, active dynamic shoulder motion and strengthening exercises were administered in the standing position until the 6^th^ postoperative month. Preservation of shoulder motion and strength was maintained for another 3 to 4 months. The patient was seen every two weeks for the first two postoperative months and was instructed and guided by us. We believe that a simple prescription of physiotherapy does not help the patient as much as this close and monitoring consultation with his surgeon.

### 2.3 Outcome Assessment

All patients were prospectively monitored for intraoperative and postoperative complications. The functional outcome was independently assessed at the third, sixth and twelfth postoperative month as well as at the last follow up using the parameters of Constant-Murley score. The average period of follow-up was 43.8 months (ranged, 24–115 months). Some patients missed one or more follow-up appointments, but all returned for the 2-year follow up visit when we also assessed the work status of those patients who had been regularly employed prior to the injury.

Standardized “trauma series” of the shoulder were ordered at presentation and at each of the follow-up appointments. We measured the impaction angle, the degree of posteromedial hinge disruption and the displacement or angulation of the greater tuberosity in the initial radiographs using the tools in the PACS system of our hospital. Progress of fracture union, loss of reduction, nonunion, residual tuberosities displacement and evidence of partial or total AVN or early osteoarthritis were assessed at each radiographic follow up control. All radiographs were measured by the senior one of us (A.P.) and by a Professor in Orthopaedics (M.T.) who was independent of the project. The mean preoperative impaction angle of the humeral head was 43,79^o^ (range, 40^o^-45^o^) and the average loss of posteromedial hinge integrity 1,29 mm (range 0-7 mm). Twenty-two patients (44.9%) had an intact hinge (0 mm of displacement), whereas the majority of the rest 27 patients (68%) showed medial displacement of the shaft in respect to the humeral head fragment.

## 3 RESULTS

The mean duration of surgery was 71 minutes (range, 61 to 102 minutes) and the average duration of hospital stay 3 days (range 2 to 5 days). The time from injury to surgery for the fresh fractures was ranged between one and four days. No intraoperative or early postoperative complications and cases of superficial or deep infection were noted. No postoperative neurovascular compromises were detected also.

### 3.1 Clinical Outcome

**Fig. (3):**
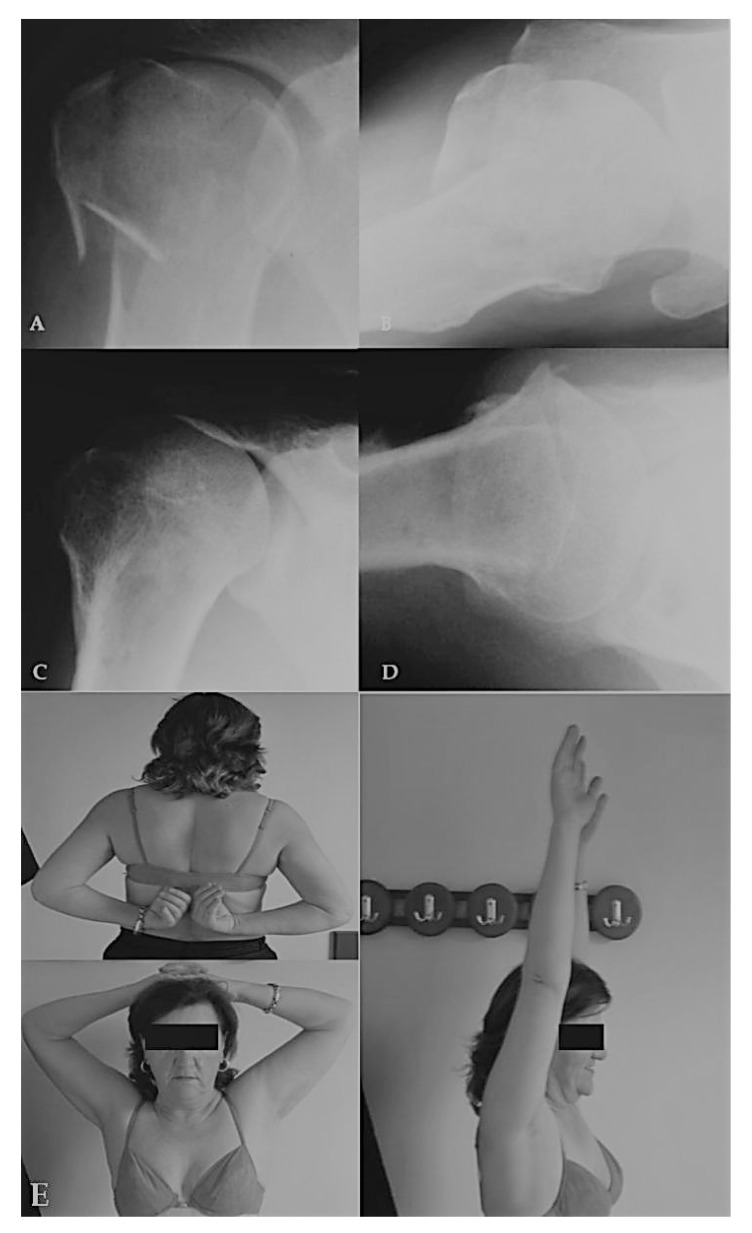
Patient N^o^ 7 with true 4-part valgus impacted fracture (**A,B**) resulted in excellent radiological (**C,D**) and clinical (**E**) outcome 26 months postoperatively.

The average Constant-Murley score of the affected shoulder was 81,7 points (range, 60 to 100) whereas the functional score as a percentage of the score in the unaffected shoulder was 86,2%. The average active elevation in a standing position was 168^o^ (160-180^o^), the average external rotation between 50^o^ and 80^o^ and the mean internal rotation with respect to the posterior spine segment reached by the thumb at least the T9 to T8. All patients were quite satisfied with the result, having no pain with vigorous activities and able to resume previous levels of daily and recreational activities (Fig. **3**). The three patients (N^o^ 23,41,43) with the AVN showed low scores prior to hemiarthroplasty whereas patient N^o^ 44 with the segmental collapse of the head was satisfied with the result as he had quite normal shoulder motion and only slight pain in vigorous activities. Patient N^o^ 45 with the nonunion had almost no motion in the shoulder before converted to RSA. All twenty-two patients who had been regularly employed prior to the injury in a sedentary job returned to their full work duties within 8 months. Of the fourteen patients who had been regularly employed in a manual job prior to the injury, 11 returned to their previous work duties.

### 3.2 Radiographic Outcome

**Fig. (4):**
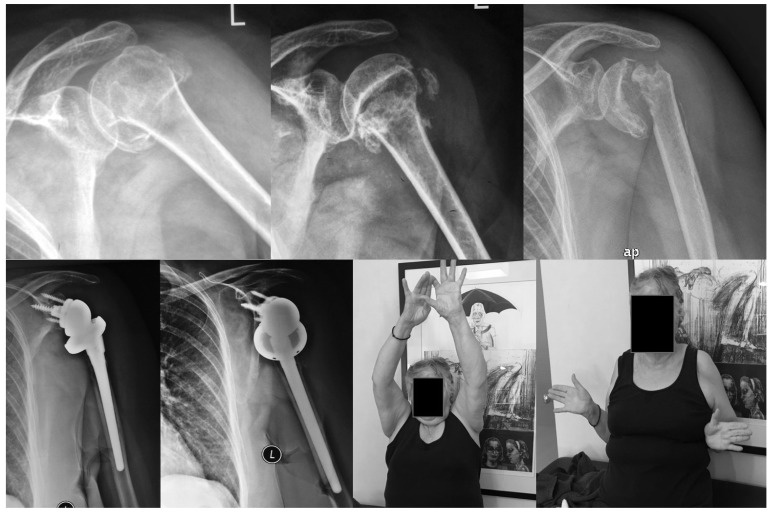
Patient N^o^ 45 was the only case with established nonunion. Due to aggressive postoperative physiotherapy the fracture was displaced leading to nonunion and a totally malfunctioned shoulder. One year postoperatively she was treated with reverse shoulder arthroplasty resulting to a moderated outcome (Constant score 60).

No cases of severe malunion, tuberosities displacement, subacromial impingement and early osteoarthritis were noted. Solid union was achieved in all except one patient within three months (range, 6 to 12 weeks). Absorption of the greater tuberosity was detected in five cases, three to four months postoperatively, without serious functional compromise of the shoulder. The degree of GTB displacement is a possible cause as in three of these cases it was displaced > 15 mm. Another possible cause is the intraoperative over-pulling of tuberosities by the surgeon as he tried to retract them below the level of the humeral head. Partial AVN of the head was seen in one patient in whom the integrity of the posteromedial hinge was disrupted 7 mm laterally. Computer tomography revealed necrosis of the lateral 1/3 of the head but the patient refused any further treatment. The only patient who developed nonunion (N^o^ 45) was living in another area and missed our regular follow up visits. She had also two operations in the spine and she had received aggressive physiotherapy in her shoulder from the second postoperative week. Due to her spine problems she presented late (after one year) with a totally malfunctioned shoulder; she was unable to move her arm and the RC tendons had disappeared. A conversion to RSA resulted to a reasonable outcome with a constant score of 69 (Fig. **4**).

**Fig. (5):**
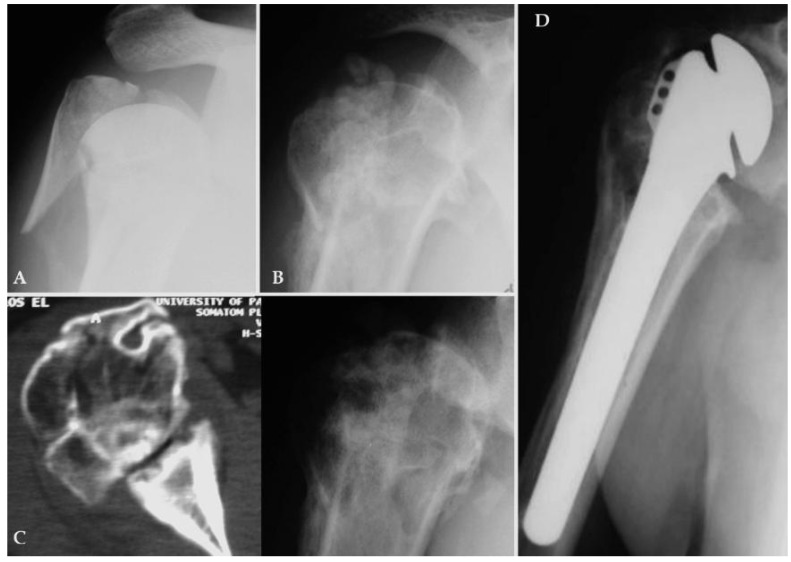
Case N^o^ 23 that was referred to our Department 30 days after the initial injury (**A**). A GTB osteotomy was performed at the time of injury leading to significant heterotopic ossification and stiffness requiring extensive capsular release one year postoperative (**B**). Two years later the diagnosis of total AVN was confirmed (**C**) and the patient was treated with shoulder hemiarthroplasty (**D**) having finally a moderate outcome 5 year after the initial injury (Constant score 75).

Special attention must be paid in our three cases with the total collapse of the head. The first patient (N^o^ 23) had an old injury (30 days old) and he referred to us by another hospital. He was initially treated conservatively with total immobilization of his shoulder. Because he was quite young, we decided to treat him by transosseous suturing despite the fact that a GTB osteotomy was considered necessary. This was an extended indication of our method and the outcome was presumable. One year later he underwent a glenohumeral joint release due to adhesive capsulitis and massive intra-articular ossifications and 3 years postoperatively a shoulder hemiarthroplasty was performed due to total collapse of the head and severe compromise of shoulder function (Fig. **5**). The second patient (N^o^ 43) didn’t conform to our rehabilitation protocol and the fracture was slightly displaced 3 weeks after the operation showing total AVN at 6 months postoperatively. Loosening of the knots might was a contributing factor to this early failure along with the fact that a fracture line through the anatomical neck was present intraoperative (the dorsomedial metaphyseal head extension was only 10 mm). A shoulder hemiarthroplasty was performed one month later resulting to an accepted outcome. The final AVN case was happened 12 months postoperative without any particular reason and it has been revised to shoulder hemiarthroplasty with a moderate outcome. Considering the pre-mentioned controversies the overall incidence of AVN and nonunion was 10.2% (5/49 cases) but only two cases could not be explained and can be attributed to the nature of the fracture.

## 4 DISCUSSION

Displaced fractures of the proximal humerus carry a significant risk of aseptic avascular necrosis of the humeral head, which is better understanding by the knowledge of the vascularization of the humeral epiphysis. The studies on this subject, and particularly those of Gerber [21], have emphasized the role of the intra-osseous anastomoses coming from the anterior circumflex humeral artery (ACA), and especially the branches of the lateral ascending artery, which runs along the lateral edge of the bicipital groove, and follows as arcuate artery after its penetration into the bone. Brooks *et al*. [22] found significant arterial anastomoses between the arcuate artery and the posterior circumflex humeral artery through vessels entering the posteromedial aspect of the proximal humerus. The arcuate artery also anastomoses with metaphyseal vessels and vessels coming from the greater and lesser tuberosities. Duparc *et al*. [23] defined the important part of the arterial blood supply carried out mainly by the posterior circumflex artery (PCA), especially to the subchondral bone of the humeral head. It is still questionable if the arcuate artery has any particular importance in the fractured humerus, because it is thought that it’s easily interrupted even in minimal displaced fractures. An investigation of the arterial supply of the humeral head in 4-part valgus impacted fractures with digital angiography and image procession, contacted by our Department [10], showed that this supply is carried out mainly from the arcuate artery and several anastomoses coming from the posteromedial capsule and also that transosseous fixation of these fractures did not seriously disrupted the vascularization of the head.

The 4-part valgus impacted fracture, as a borderline lesion in the continuum of head translation according to Neer [2], is expected to have a lower incidence of AVN because some of the posteromedial neck vessels may be preserved if the integrity of the medial hinge remains and the translation of the humeral head is zero or minimal. Maintenance of this medial hinge may also help in fracture reduction, since it serves as a support point (fulcrum) for the humeral head to return to its varus position, without losing contact with the metaphyseal region of the diaphysis. Hertel *et al*. [24] has investigated several predictors of humeral head ischemia after intracapsular fractures of the proximal humerus by observing the perfusion backflow after drilling a borehole into the humeral head center; the authors proposed several risk factors for AVN such as complex fractures with 3 or 4 fragments, dislocation of the head or splitting intra-articular components but to their opinion the most relevant predictors of ischemia are the *length of dorsomedial metaphyseal extension*, the *integrity of the medial hinge* and the *basic type of the fracture*.

Current trends in surgical reconstruction of 4-part valgus impacted fractures include limiting soft tissue dissection, reduction (or limited elevation) of the head to its anatomical position and internal fixation using tubular plates, locking plates, percutaneous pinning, screw-wiring techniques or transosseous sutures with the additional use of bone, synthetic or structural grafts in some reports [1, 4, 10-18, 25-30]. Apart from the risks for AVN, loss of reduction, malunion and hardware-associated complications, such as breakage, migration, joint or neurovascular penetration can also occur [31-33].

Jakob *et al*. [1] reported an incidence of AVN up to 26% on 19 patients who underwent closed reduction and percutaneous KW fixation (5 patients) or open reduction and minimal internal fixation with KW, screws, or KW and cerclage wires (14 patients). Resch *et al*. [15] reported an AVN rate of 9% on 22 patients who underwent internal fixation with KW, autogenous cancellous bone graft augmentation and supplementary transosseous fixation of the tuberosities. The same author in 1997 [34], reported on 13 patients using a closed technique of percutaneous reduction, KW and screw fixation; one patient developed AVN, a rate of 7.6%. Yu *et al*. [12] reported excellent results without any case of AVN on nine 4-part valgus impacted fractures treated with the screw-wiring technique and autogenous bone grafting. Hockings and Haines [13] followed 11 patients that have been treated with a minimal invasive technique of transosseous suturing without any use of bone grafting or hard material. The results were quite good and the rate of AVN 9%. Robinson *et al*. [25] reported on 25 patients with 4-part valgus fractures treated with open reduction, filling of the metaphyseal cavity with Norian SRS and internal fixation with isolated screws or buttress plates. No patient had signs of osteonecrosis on the latest follow-up and the mean Constant score was 80 at one year. Atalar *et al* [17] reported on 10 patients treated with head elevation, transosseous sutures and bone grafting; the rate of AVN was 8.3% and the CS 81.5. The same group of authors [30] reported recently a technique of structural graft augmentation of the elevated head and fixation with locking plates in 10 patients; no cases of head collapse or AVN were noted after a mean follow up period of 22.5 months. Keener *et al* [35] and Bogner *et al* [14] reported on 12 and 16 patients in respect utilizing a percutaneous technique and fixation with KW and/or cannulated screws; AVN rates were 8.3% and 18.75% respectively. Two previous reports from our Department [10, 19] on 15 and 45 patients in respect showed a mean AVN rate of 6.6% and 11% accordingly. The clinical and radiological results of most of the above reports are summarized in a recent systematic review conducted from our Department [18], which was based on 12 eligible studies including 190 four-part valgus impacted fractures in 188 patients. One of the most important finding in this review was that the overall rate of AVN was similar in both ORIF and percutaneous Least Possible Fixation Techniques regardless the follow up period, the surgical approach and the fixation method.

In general, the use of heavy non-absorbable transosseous sutures is an attractive choice for fixation of 4-part valgus impacted fractures. After a midterm follow up of 43.8 months on average, we had four cases of AVN (8.1%) and one established nonunion (2%). The functional Constant score was 86.2% and almost all patients were satisfied with the result. The powerful characteristics of this study is the adequate patients sample, the long-term follow up and the well investigated clinical and radiological outcome but some limitations still exist: First, we attribute our good results to careful surgical technique, especially the solely use of osteosutures and minimal soft tissue dissection. Precise assessment of these surgical details is difficult. Second, the repeatability of radiographic measurements is lacking standardized methods to document as the humeral head is often either internally or externally oriented, depending on the radiograph plate and beam. An effort was made in all cases to obtain AP views in zero rotation and estimate the radiological parameters using special tools in our online PACS environment. Finally, some controversies exist about the consequences to normal shoulder anatomy by the minimally reduced humeral head. A biomechanical investigation may help in the future to combine the mechanical parameters with the long term clinical outcome.

Despite these limitations, we recommend the use of open reduction and minimal internal fixation of 4-part valgus impacted fractures of the proximal part of the humerus in an attempt to achieve a satisfactory and stable reduction, adequate rotator cuff repair and immediate shoulder joint motion avoiding the complications of any hard material application.

## CONCLUSION

Advantages of this minimally invasive technique can be summarized to shorter operative time, no use of hardware, minimal soft tissue damage, low incidence of avascular necrosis, stable osteosynthesis with “tension band effect” and adequate rotator cuff repair allowing for early joint motion.

## Figures and Tables

**Table 1:** Overview of clinical and radiological data.

Patient	Age,	Side	Type of Injury	Associated Pathology or Injuries and Delay	Valgus Impaction Angle (^o^)	Medial Hinge Integrity (mm)	Follow up (months)	Constant Score [injured / normal shoulder]	Complications
	Gender			in treatment					
**1 1. NP**	59,F	R	FS	21 days old	42	1 (L)	24	87 (95)	–
**2. BB**	51,M	R	FS	–	45	0	28	90 (100)	–
**3. AS**	47,F	R	FS	–	42	0	25	85 (95)	–
**4. MA**	35, F	R	TA	–	44	3 (M)	24	88 (92)	–
** 5. GG**	61,F	L	FS	–	45	1 (M)	28	75 (88)	–
**6. MN**	55,M	R	FH	–	44	1 (L)	28	90 (95)	GTB absorption
**7. MD**	43, F	R	FS	–	45	0	26	95 (100)	–
**8. PX**	29,M	L	TA	Clacaneous	45	0	26	100 (100)	GTB absorption
				fract. (R)					
**9. MA**	45,M	R	TA	Radial head	45	2 (L)	72	70 (90)	–
				fract. (R)					
**10. KA**	32,F	L	TA	–	45	0	26	86 (95)	–
**11. PT**	55, F	R	FS	–	42	0	62	95 (98)	–
**12. MA**	68,M	R	FS	–	45	0	68	90 (100)	–
**13. DI**	32,M	L	TA	–	42	2 (M)	60	80 (92)	–
**14. PB**	56,F	R	FS	Ankle fract. (L)	45	0	40	90 (95)	–
**15. DN**	62,F	R	FS	–	42	0	45	75 (90)	–
**16. PE**	66,F	R	FS	–	44	1 (M)	42	76 (88)	–
**17. KI**	45,F	L	FS	–	45	0	43	82 (90)	–
**18. DE**	50,F	R	FS	–	45	4 (M)	46	80 (90)	–
**19. SD**	45,F	L	FS	–	44	3 (M)	47	85 (95)	GTB absorption
**20. RG**	65, F	R	FS	–	43	1(M)	37	70 (88)	–
**21. SK**	58,F	L	FS	–	45	3 (M)	44	90 (87)	–
**22. GM**	73,F	R	FS	–	44	1 (L)	59	75 (82)	–
** 23. KE**	34,M	R	TA	30 days old	44	1 (M)	50	65 (88)	AVN (SH 8 months pop)
** 25. AA**	34,M	R	TA	–	45	0	50	75 (86)	–
**24. KN**	55,M	L	FS	–	45	0	50	60 (89)	–
**26. TE**	47,F	L	FS	–	45	0	28	90 (96)	–
**27. SG**	21,M	R	TA	–	44	0	53	80 (95)	–
**28. ZE**	45,F	L	FS	–	43	0	52	100 (100)	–
**29. MN**	42,F	L	FS	–	45	0	56	100 (98)	–
**30. NM**	67,F	R	FH	Upper cervical spine injury	45	2 (M)	55	90 (94)	–
**31. PE**	68,F	R	FS	–	45	0	54	85 (90)	–
**32. SB**	63 F	L	FS	–	43	2 (M)	60	80 (87)	–
**33. KA**	48,M	R	FH	–	45	2 (M)	58	90 (92)	–
**34. NG**	26,M	R	TA	Wrist fracture (L)	44	1 (L)	60	90 (100)	–
**35. XZ**	71,F	R	FS	–	41	2 (M)	53	85 (95)	–
**36. KA**	23,F	R	FS	16 days old	45	2 (M)	60	80 (92)	–
**37. TA**	47,M	L	TA	–	44	0	65	87 (95)	–
**38. FG**	69,M	R	FS	–	40	3 (M)	66	86 (97)	–
**39. AI**	62,F	L	FS	–	41	4 (L)	72	85 (92)	GTB absorption
**40. TS**	51,F	L	FS	22 days old	42	2 (M)	65	65 (90)	–
**41. KM**	66,M	R	FS	–	45	0	25	60 (88)	AVN (SH 1 year pop)
**42. M N**	63,F	R	FS	4-part fracture-dislocation [R]	43	3 (M)	115	90 (90)	Partial AVN (no further treatment)
**43. SB**	67,F	R	FS	–	42	3 (L)	30	67 (89)	AVN (SH 6 months pop)
** 44. MF**	61,F	R	TA	Tibial plateau fracture (R)	43	7 (M)	56	75 (92)	–
**45. KG**	65,F	L	FS	–	44	4 (M)	65	60 (89)	Nonunion (converted to RSA 1 year pop)
**46. GB**	82,F	R	FS	–	45	3 (M)	36	87 (92)	–
**47. AE**	53,F	L	FS	–	40	1 (M)	34	85 (90)	GTB absorption
**48. MP**	73,F	R	FS	–	45	0	24	84 (87)	–
**49. KG**	71,F	R	FS	–	45	2	36	81 (83)	–
**FS:** simple fall, **FH:** fall from height, **TA:** traffic accident, **AVN:** avascular necrosis, **GTB:** greater tuberosity, **SH:** shoulder hemiarthroplasty, * M and L means the direction of shaft displacement in respect to the humeral head									
